# Extrusion-Based 3D Bioprinting of Gradients of Stiffness, Cell Density, and Immobilized Peptide Using Thermogelling Hydrogels

**DOI:** 10.1021/acsbiomaterials.1c00183

**Published:** 2021-05-10

**Authors:** Merve Kuzucu, Grace Vera, Marco Beaumont, Sascha Fischer, Pan Wei, V. Prasad Shastri, Aurelien Forget

**Affiliations:** † Institute for Macromolecular Chemistry, University of Freiburg, Stefan-Meier-Str. 31, 79104 Freiburg, Germany; ‡ School of Chemistry and Physics, Queensland University of Technology, 2 George St, Brisbane City, Queensland 4000, Australia; § Institute of Chemistry of Renewable Resources, University of Natural Resources and Life Sciences (BOKU), Konrad-Lorenz-Straße 24 3430 Tulln, Austria; ∥ BIOSS, Centre for Cell Signalling Studies, Schänzlestr. 18, 79104 Freiburg, Germany

**Keywords:** agarose, polysaccharide, anisotropy

## Abstract

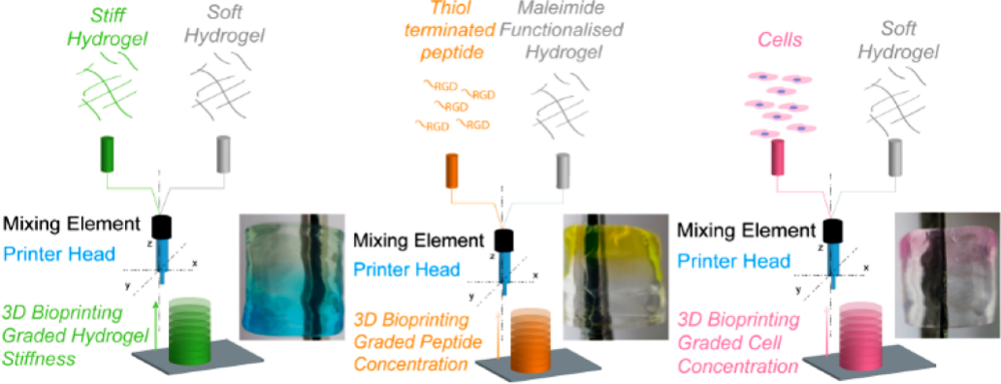

To study biological processes *in vitro*, biomaterials-based engineering solutions to reproduce the gradients observed in tissues are necessary. We present a platform for the 3D bioprinting of functionally graded biomaterials based on carboxylated agarose, a bioink amendable by extrusion bioprinting. Using this bioink, objects with a gradient of stiffness and gradient of cell concentration were printed. Functionalization of carboxylated agarose with maleimide moieties that react in minutes with a cysteine-terminated cell-adhesion peptide allowed us to print objects with a gradient of an immobilized peptide. This approach paves the way toward the development of tissue mimics with gradients.

To reproduce the organization of mature and diseased tissue, materials that can copy the graded architecture observed in natural tissues are needed.^1,2^ Some tissues like the osteochondral interface are organized into graded architecture. Several approaches have been proposed to reproduce biological gradients,^3^ such as surface coating,^4^ ice templating for gradient of porosity,^5^ selective irradiation using masks,^6^ and recently additive manufacturing.^1,7−9^ Different methods have been proposed for the bioprinting of functionally graded biomaterials, for instance, light activated immobilization techniques,^10^ extrusion printing with alginate,^11^ and mixing of gelatin methacrylate.^12^ While the light activated techniques offer high resolution and speed of patterning, it is challenging to change the cell concentration during manufacturing. Extrusion techniques based on alginate were used to achieve a gradient of hydroxyapatite particles^11^ and a gradient of cell type, whereas methods based on gelatin methacrylate were used to make a 2D gradient of stiffness and cell type.^12^ Bioprinting of multiple materials into one small printing nozzle is quite challenging as one needs to overcome the laminar flow that stops liquids from mixing.^13^ Several approaches have been proposed to overcome this phenomenon. Microfluidic systems can be designed in many shapes, and they allow the reduction of the volume needed for extrusion, thus increasing the printing resolution.^14^ Using a microfluidic printhead allows precise control of the composition of the extruded materials.^15^ As an alternative approach to microfluidics, mechanical mixers have been used for gradient printing; for instance, active mixers were used to print silicone-based elastomeric ink in which the concentration of a fluorescent pigment is continuously changed during printing.^16^ However, when mixing mammalian cells, the challenge is to obtain a homogeneous bioink while not inducing a high shear stress during printing that is detrimental for the cells.^17^ An alternative to active mixers is static mixers, which have been used to mix cellulose-based biomaterials to create a gradient of stiffness,^18^ and mixing of a two-part bioink composed of hyaluronic acid and carboxymethyl chitosan loaded with fibroblast.^19^

Mixing during printing requires a system where the bioink viscosity, extrusion flow rate, printing speed, cell concentration, and reaction kinetic of the molecules to be immobilized are all compatible for the selected bioprinting technique. There are no previous reports that have shown one method that offers the possibility of 3D bioprinting gradients of stiffness and cell concentration and immobilized signals, which is the degree of complexity needed to precisely reproduce anisotropic tissues.^7^

The bioinks employed in this study are based on our carboxylated agarose (CA) bioink platform (Figure 1A).^20−23^ Upon controlled oxidation of the polysaccharide backbone, a switch in the secondary structure of agarose from the α-helix to the β-strand is observed.^24^ This change of secondary structure modifies the polymer backbone organization and ultimately the mechanical properties of the resulting hydrogel. Controlling the number of carboxylic acid groups along the polymer backbone provides a means to precisely tune the hydrogel stiffness. This same tunability of the mechanical properties of the hydrogels can also be achieved by blending the native agarose with fully carboxylated agarose.^25^ This enables us to obtain hydrogels of varying stiffness without the need to change the concentration and viscosity of the hydrogel precursor solutions.^22^ This property has allowed us to bioprint objects composed of domains of discrete stiffness using a drop-on-demand bioprinting process.^22^ CA can be printed either in gel state due to its shear-thinning properties^23^ or in the solution state; the latter is the method of choice in the case where CA is modified inline during printing, e.g., to chemically attach biological signals. While we have optimized the printing of the CA hydrogel precursor for drop-on-demand bioprinting,^22^ we first optimized the bioink parameter using a custom-made extrusion-based setup (Figure 1B,C) that can mix bioinks during printing. Similarly to what was reported in various studies for the mixing of two bioinks,^18^ we used a static mixer that we positioned before the printing nozzle (SI Figure 5). On this platform, we bioprinted planar serpentine and cylindric shapes (SI Figure 1). We found out that for a CA of different rheological properties, the best bioprinting was obtained with 6% w/v with shear moduli (at 1 Hz) of 1520 ± 106 Pa for the soft bioink, 2234 ± 149 Pa for the medium, and 3745 ± 93 Pa for the stiff one (SI Figure 2).

**Figure 1:**
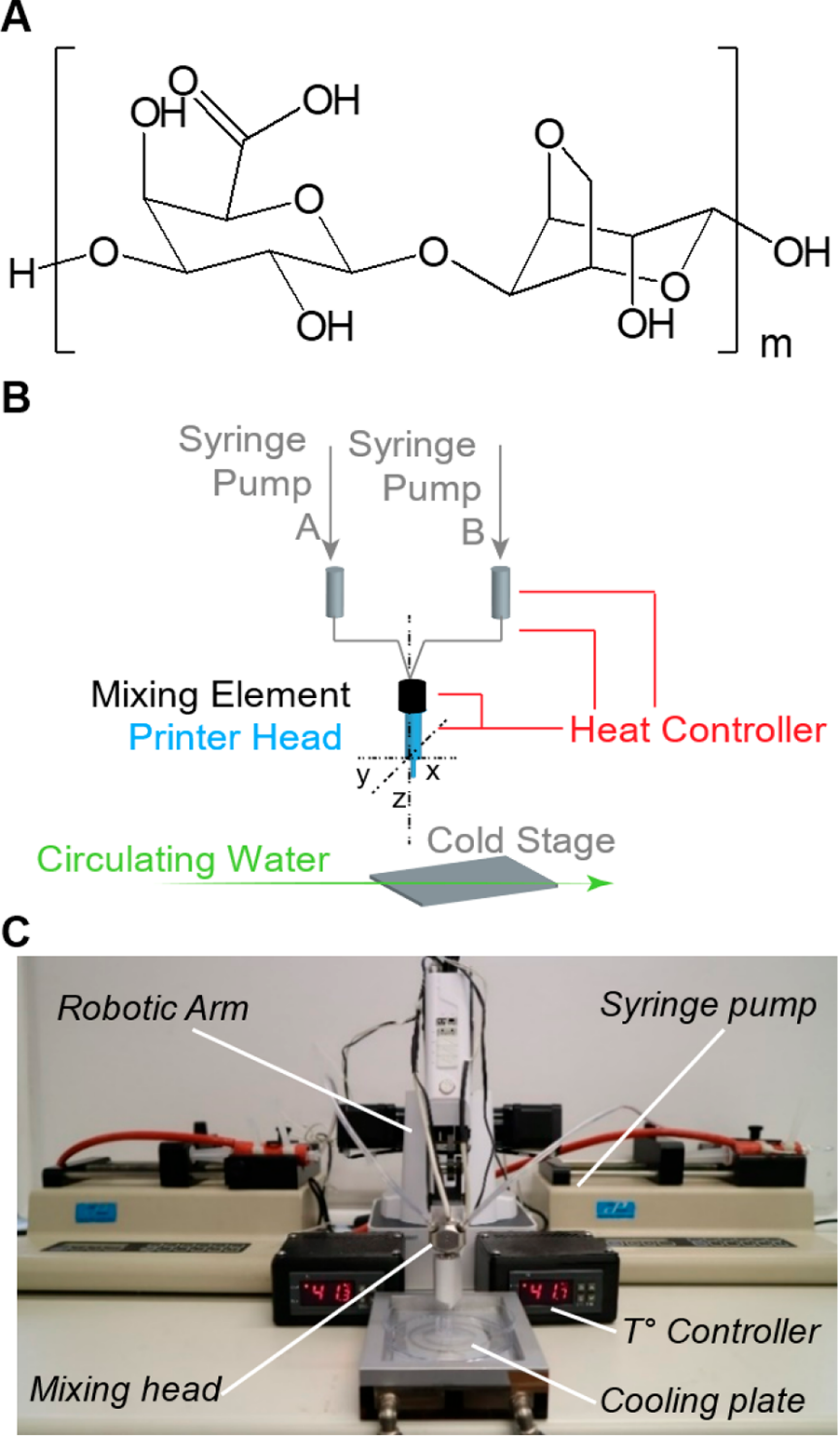
Bioprinting setup. (A) Chemical structure of thermogelling carboxylated agarose bioink. (B) Schematic of our custom-made printing platform based on two syringe pumps extruding the different components of the bioink into a temperature-controlled mixing printing head. (C) Photograph of the custom-made print setup based on a robotic arm that prints the materials on a cooled stage.

Previously, we have reported that the mechanical properties of CA hydrogels can be precisely tuned from 10 to 1 kPa by blending fully carboxylated agarose with native agarose.^25^ This implies that in theory the elastic properties of a bioprinted hydrogel can be tailored by mixing CAs of various stiffness using the optimized bioprinting parameters for the three CAs (soft, medium, and stiff). Using the two-ink mixing printing head (SI Figure 3), CAs of different stiffnesses can be ad-mixed during printing by controlling the set flow rate of each of the two CA bioink components to create bioprinted objects with a gradient of stiffness. Once extruded, the bioink forms a gel on a water-cooled printing stage (SI Figure 4).

This approach was first tested in a 2D layout (Figure 2A) and then with the cylinder configuration (Figure 2B). As a first approximation, to visually follow the CA ratio in the print (and the stiffness gradient), the soft CA was colored in blue, the medium CA in green and the stiff CA in red. The use of colored inks facilitated the optimization of the printing process and enabled online adjustments. During printing of the serpentines, the flow rate of the two inks (stiff and soft, medium, and soft) was varied (SI-Table 1) to obtain a single line with a gradient of stiffness (Figure 2C and D). Bioprinting of cylinders was conducted analogously to yield hollow cylinders with graded stiffness from soft to medium (Figure 2E) and from soft to stiff (Figure 2F). The graded stiffnesses in the 2D (Figure 2G) and 3D (Figure 2H) bioprinted hydrogel objects were validated by indentation tests, and the elastic modulus was extracted from these measurements. In the serpentine shape, both graded prints led to a broad range of elastic moduli spanning 103 kPa for the medium CA and 139 kPa for the stiff CA. The gradual introduction of the soft CA into the mixing head led to a drop in elastic modulus to 38 and 56 kPa for the medium/soft and stiff/soft mixtures, respectively. In the 3D cylinder shape, indentation tests revealed a broader range of elastic moduli for the stiff/soft from 258 to 57 (±19) kPa. Conversely, the medium to soft graded cylinder led to a shorter range of compressive modulus from 85 to 49 (±11) kPa. In general, the softer parts (blue color) of the different objects (2D and 3D) were of comparable modulus range (38–57 kPa), whereas the upper limit of the modulus (stiff or medium) was strongly dependent on the print type. The 3D stiff/soft object also had a significantly higher elastic modulus and the 3D medium/soft one a significantly lower one than compared to the 2D objects. Potential explanations for this discrepancy can be (a) the higher total extruded volume in 3D objects and hence a less steep and homogeneous gradient, and (b) the fact that 3D objects are composed of several printed layers in the direction of the print that is softer-to-stiffer, versus stiffer-to-softer, can impact the gelation in these layers, thus influencing the measured moduli. This limitation may also arise from the dead volume in the mixing head. A smaller mixing and extrusion head should lead to a higher resolution of the mechanical gradient. However, this work represents a first demonstration of the capability of CA hydrogels to yield 3D-printed objects with graded mechanical properties by extrusion printing.

**Figure 2:**
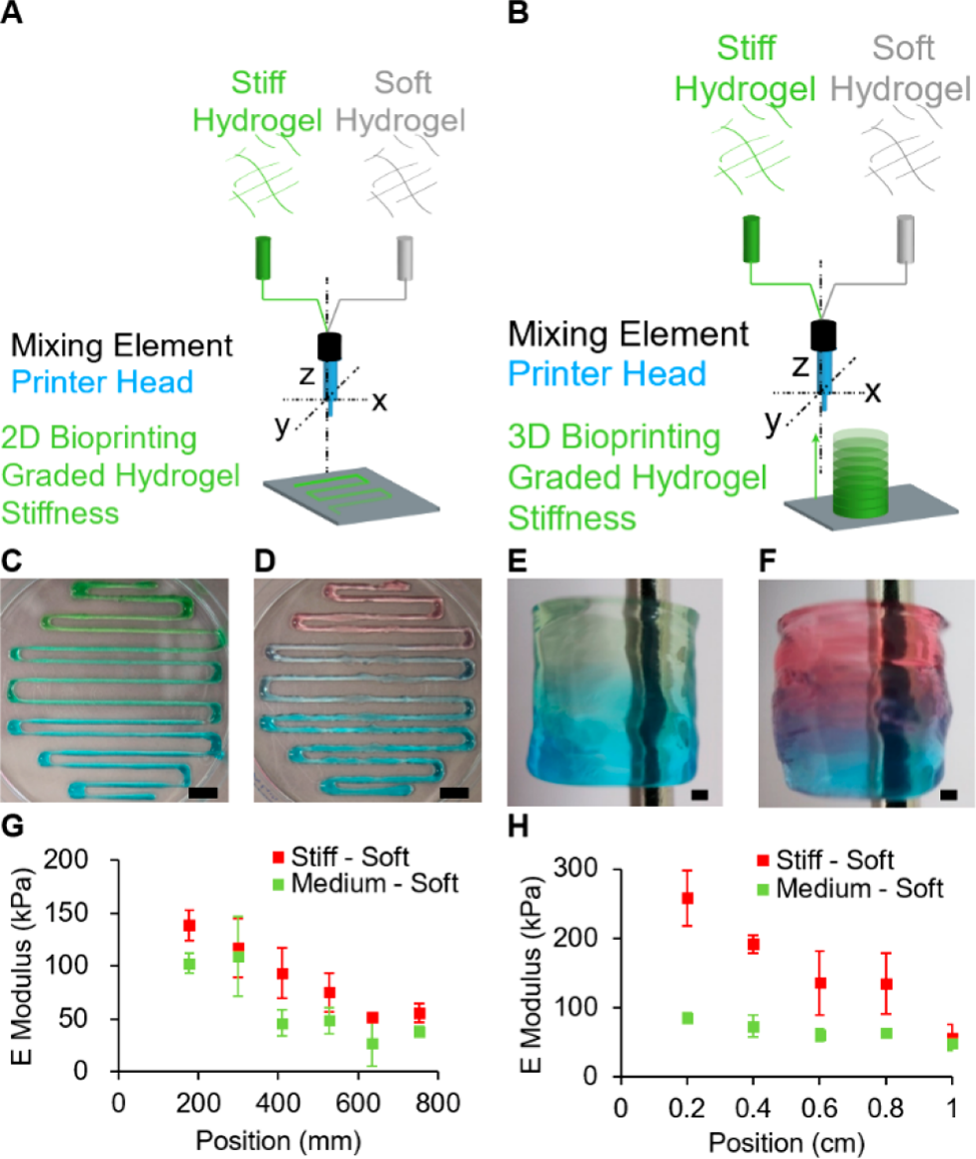
Gradient of stiffness. Scheme depicting the bioprinting experiment of (A) 2D and (B) 3D graded stiffness. Colored gel reveals the change in hydrogel composition from (C, E) stiff (red) to soft (blue) and (D, F) medium (green) to soft (blue). The mechanical properties of (G) 2D and (H) 3D prints were analyzed by indentation tests; the E modulus equals the indentation elastic modulus. Error bars show a standard deviation for *n* = 3. Scale bars: (C, D) 10 mm, (E, F) 1 mm.

While gradients of stiffness have been observed in several mature and diseased tissues, the anisotropic extracellular composition of proteins and polysaccharides is also a characteristic of the tumor tissue^26,27^ and osteochondral interface.^28^ To reproduce such an anisotropic ECM composition, one must be able to immobilize biological signals that mimic the cell–ECM interface. In that matter, cell-adhesion peptides (CAPs),^29^ such as the integrin-binding RGDSP sequence, are ubiquitously known to mimic fibronectin.^30^ To generate an online gradient of immobilized signals, the immobilization of CAP must be controlled during the printing. To achieve this, a chemical click reaction that allows rapid covalent binding of the peptide onto the CA polysaccharide backbone is required. Thiol–ene Michael addition reactions between maleimides and thiols were chosen here. It is a rapid and biocompatible reaction, and furthermore, the thiol functional group can be easily incorporated into a peptide sequence by introducing a cysteine amino acid residue at the end of the sequence.^31^

Toward this end, CA was first functionalized through carbodiimide coupling chemistry to introduce a maleimide functionality onto the CA backbone (Figure 3A). This was verified by FTIR (SI Figure 6A), and we calculated by NMR (SI Figure 6B) that 2.5% of the repeat units were modified with maleimide groups. The maleimide-CA was then reacted with thiol-terminated peptide (CRGDS) in under 12 min (Figure 3B). The precision of the peptide immobilization during printing is a combination of two factors: (1) peptide diffusion in the hydrogel and (2) kinetics of the chemical reaction. While the CRGDS is water-soluble, it has only two residues with a high hydropathy index, which could be limiting the peptide diffusion. Therefore, the thiol–ene kinetics are suitable to immobilize the CAP onto the bioink preventing its uncontrolled diffusion. To conserve the potential of CA as a cell culture substrate, the addition of the maleimide onto the CA backbone needs to have a limited impact on the hydrogel’s mechanical properties. After modification, we studied the secondary structure by circular dichroism and could not observe differences between CA and functionalized CA (SI Figure 6C). Rheological studies confirmed that the shear modulus of the functionalized hydrogel was within the same order of magnitude as the native soft CA (Figure 3C), for a 2% w/v hydrogel 183 and 138 Pa, respectively.

**Figure 3:**
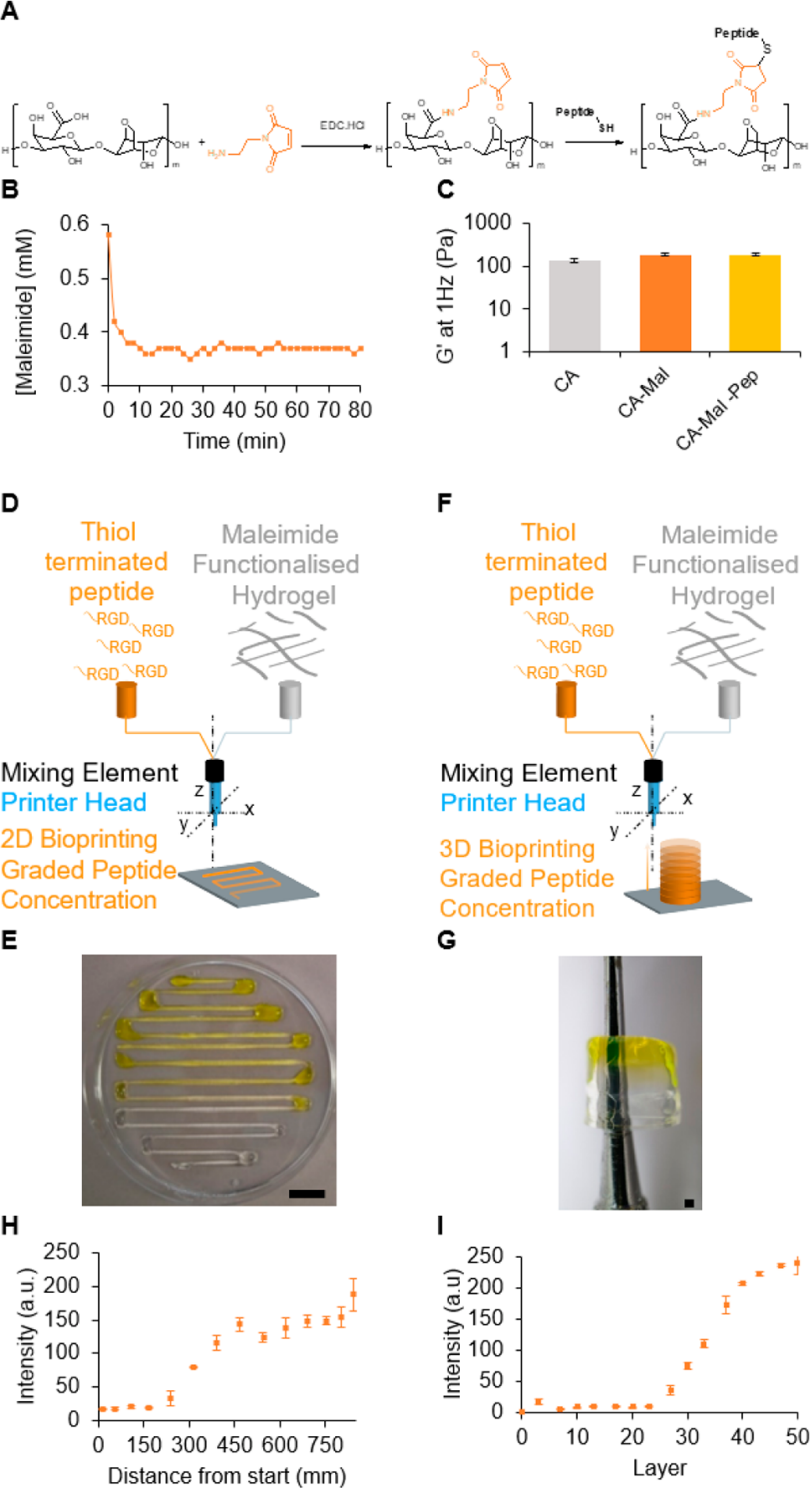
Gradient of peptide concentration. (A) Chemical reaction used to functionalize the soft carboxylated agarose hydrogel with maleimide and successive click reaction with a cysteine-terminated peptide. (B) Reaction kinetics of functionalized agarose with the CRGDS peptide. (C) Effect of the functionalization on the rheological properties is shown through the storage shear modulus of native soft carboxylated agarose and its derivatives. Scheme representing the (D) 2D and (F) 3D bioprinting experiment of graded peptide immobilization. (E and G) Soft-maleimide bioink prints with CRGDS-FITC. Color analysis showing the change in intensity across the printed (H) 2D object and (I) 3D object. Error bars are standard deviation for *n* = 3. Scale bars: (E) 10 mm, (G) 1 mm.

To visualize the peptide gradient, the maleimide functionalized CA was mixed during printing with a solution of FITC-labeled CRGDS and used in combination with a maleimide functionalized CA to print 2D serpentine shapes with a gradient of peptide concentration (Figure 3D). Increasing the flow rate of the peptide solution during printing (SI Table 3), resulted in the extrusion of an ink with a higher peptide concentration as seen by the increase of the yellow color intensity in the hydrogel, which is characteristic of the FITC dye (Figure 3E). Similarly, increasing the flow of the peptide solution (SI Table 4) during printing of the 3D cylinder with the soft maleimide-functionalized CA ink (Figure 3F) resulted in an increase of the FITC yellow color across the *z* axis of the object. This demonstrates the graded concentration of the FITC-functionalized peptide in the object (Figure 3G). The chemical anisotropy of the printed objects was characterized by measuring the intensity of the yellow color across the printed serpentine 2D object (Figure 3H) and the 3D cylinder (Figure 3I). This suggests that a gradient of CRGDS had been established within the printed structures. Validation of the covalent immobilization of the peptide in the bioprinted structure was performed by equilibration of the printed objects with CA and maleimide-CA in deionized water. The supernatant was removed at different time intervals, and the concentration of peptide in the supernatant was measured using a UV–vis spectrometer. While the yellow color associated with FITC-CRGDS was completely washed out after 6 h from the structures printed with unmodified CA ink, no release of the FITC-CRGDS was observed in the structures printed using the maleimide-CA ink (SI Figure 6D). This demonstrates that the CRGSP peptide is covalently immobilized in the CA hydrogel functionalized with maleimides. These results demonstrate that maleimide-based thiol–ene Michael addition can be used to create a gradient of immobilized biological signal in extrusion bioprinting.

In some tissues, anisotropy manifests itself also as a gradient of cell density.^8^ To reproduce such cellular anisotropy, during the printing step, a varying concentration of cells needs to be introduced in the bioink. Using the same setup, we tested the incorporation of a solution of human embryonic kidney (HEK-293) cells into the soft CA bioink in the 2D serpentine shape (Figure 4A). The CA bioink was formulated in PBS and the cells suspended in phenol red supplemented media, thus allowing for the visual tracking of the printed gradient (Figure 4B). As previously reported on drop-on-demand,^22^ and extrusion bioprinting,^21^ incorporation of cells within the ink was possible (Figure 4C and D). To quantify the cellular gradient, immediately following printing, cells were stained with Hoechst dye (live cells stain blue) and imaged using a fluorescent microscope (Figure 4E and F). The number of cells per field of view as a function of the length of the serpentine structure (Figure 4G) or per deposited layer of the cylinder was counted (Figure 4H). While some local variation in cell density was observed in the serpentine shape gradient, a clear increase in cell density from zero cells at the beginning of the serpentine to 2500 cells per field of view was observed, and in the cylinder an increase from zero to 40 cells per layer was observed. Since the two designs were geometrically complementary, these results demonstrate the possibility to create gradients in *x*, *y*, and *z* axes. Finally, we assessed the cell viability after extrusion, and we report this value as a percentage normalized to cells suspended manually in the hydrogel (Figure 4I). It was demonstrated that the shear stress plays a critical role in cell viability during extrusion bioprinting, and this parameter is a function of the bioink flow, bioink viscosity, and nozzle diameter.^17^ Therefore, we present here the percentage of viable cells as a function of the flow rate. In CA, cells do not physically attach to the polysaccharide, and we observe here only the impact of the extrusion as normalized to the cells suspended in the matrix without adhesion factors. At a low flow rate, we did not observe a difference in cell viability as compared to the cell manually seeded in the hydrogel (Figure 4J). As predicted, for higher extrusion mixing (above 0.02 mL/min), we observed a high loss of cell viability (Figure 4K). While this demonstrates that with a simple static mixer, we can bioprint a gradient of cell density, further studies on the impact of the mixer shape, flow rate, and cell adhesion to the bioink are required to improve the cell survival during mixing.

**Figure 4:**
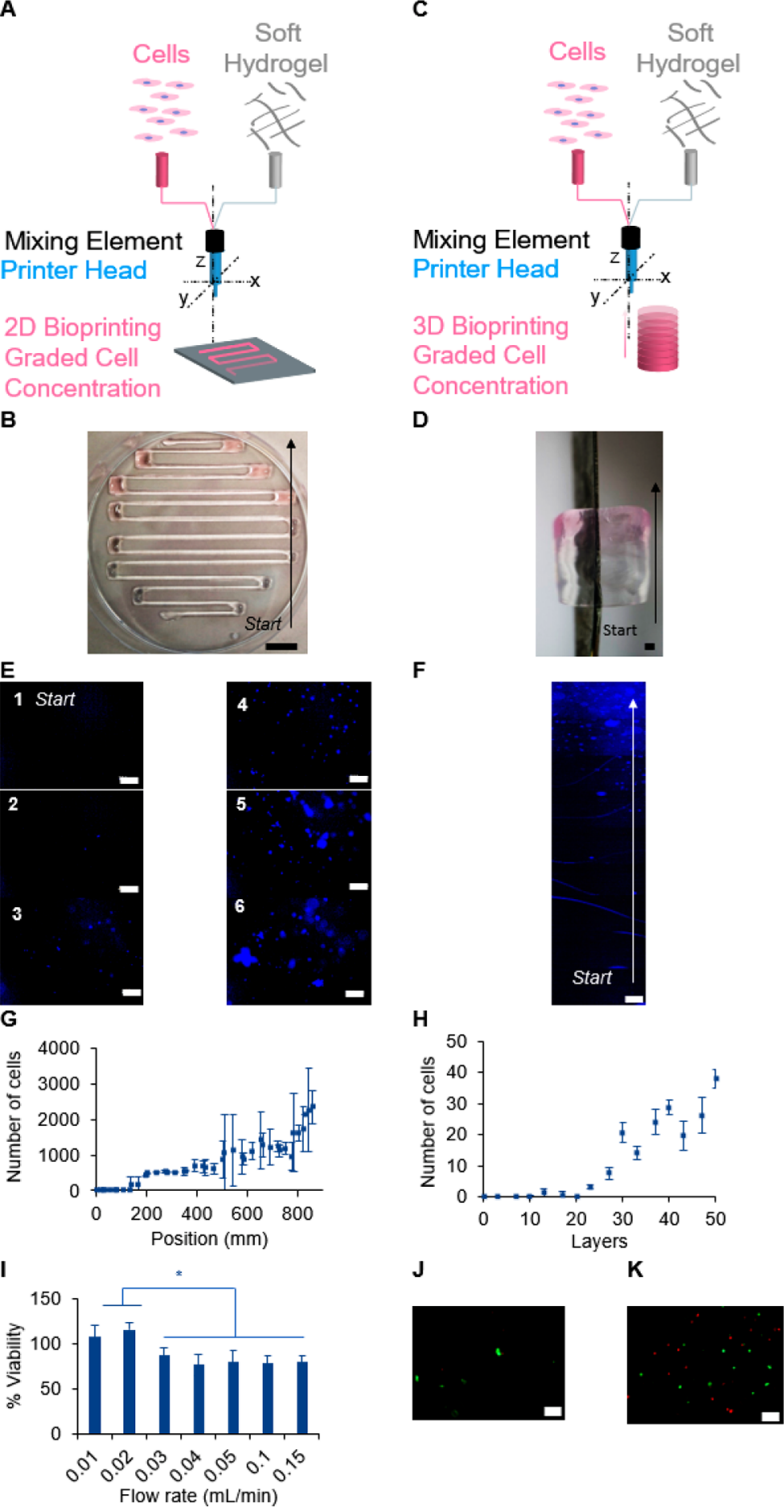
Gradient of cell concentration. Scheme representing the (A) 2D and (C) 3D bioprinting experiment of graded concentration of human embryonic kidney cells (HEK-293). (B and D) Printed 2D and 3D objects with a gradient of HEK-293 cells. Cells were stained with Hoechst dye (blue color) to visualize them by microscopy in the (E) 2D serpentines and (F) the lateral side of the 3D object. The number of cells at different positions of (G) 2D object and (H) 3D cylinder. Error bars represent the standard deviation for *n* = 3. Scale bars: (B) 10 mm, (D) 1 mm, (E and F) 200 μm. (I) Cell viability as a function of the flow rate normalized to cell viability dispersed in the hydrogel, *n* = 3, * for *p* < 0.05, unpaired *t* test. Representative microscopic image by LIVE/DEAD assay for a flow rate of 0.01 mL/min (J) and 0.15 mL/min (K). Scale bar 100 μm.

This work presents a first proof-of-concept of a method capable of bioprinting objects with a gradient of stiffness and a gradient of immobilized peptides and a gradient of cell density. The gradients were here achieved independently from each other. We envision that this method could be used for the biofabrication of objects that combine all three gradients to mimic complex anisotropic tissues such as the osteochondral interface. Further work on the static mixer shape should enable improvement of the cell viability and precision of each type of gradient.

## Supporting Information Available

Materials and methods, optimization studies of the bioink formulation, scheme of the bioprinter, extrusion parameter tables, and CAD files (PDF)

## Abbreviations

CA: carboxylated agarose
CAP: cell adhesion peptide
