# Supplementary material for: Extrusion-Based 3D Bioprinting of Gradients of Stiffness, Cell Density, and Immobilized Peptide Using Thermogelling Hydrogels
Source: ACS Biomater Sci Eng. 2021 May 10;7(6):2192–7. doi: 10.1021/acsbiomaterials.1c00183 (PMC8207502; doi:10.1021/acsbiomaterials.1c00183)
Supplement: Supplementary file 1 — ab1c00183_si_001.pdf [file ab1c00183_si_001.pdf]

Supporting Information

Extrusion-based 3D bioprinting of gradients of stiffness, cell density and immobilized peptide using thermogelling hydrogels

Merve Kuzucu[#], Grace Vera[#], Marco Beaumont[†,$], Sascha Fischer[#], Pan Wei[#], V. Prasad Shastri[#,‡] and Aurelien Forget[#]*

#Institute for Macromolecular Chemistry, University of Freiburg, Stefan-Meier-Str. 31, 79104 Freiburg, Germany

†School of Chemistry and Physics, Queensland University of Technology, 2 George St, Brisbane City, QLD 4000, Australia

$Institute of Chemistry of Renewable Resources, University of Natural Resources and Life Sciences, (BOKU), Konrad-Lorenz-Straße 24 3430 Tulln, Austria

‡BIOSS, Centre for Cell Signalling Studies, Schänzlestr. 18, 79104 Freiburg, Germany

* Correspondence to: aurelien.forget@makro.uni-freiburg.de

Table of Contents

Materials and Methods....3

Carboxylated agarose....3

3D bioprinter....3

Bioprinting design....3

3D bioprinting optimization....3

Maleimide functionalized agarose....4

^{1}H-NMR spectroscopy....4

FTIR spectroscopy....4

CD spectroscopy....4

UV-Vis spectroscopy kinetic....4

Rheological measurements....5

Bioprinting of stiffness gradient....5

Bioprinting of peptide gradient....5

Cell concentration gradient bioprinting....5

Figure S1. CAD of the printed objects....7

Figure S2. Optimization of printing parameter....8

Figure S3. Technical drawing of the mixing head....9

Figure S4. Technical drawing of the cooling stage....9

Figure S5. Static mixer....10

Table S1. Flow rate parameters for 2D Bioprinting of graded mechanical properties....11

Table S2. Flow rate parameters for 3D Bioprinting of graded mechanical properties....11

Table S3. Flow rate parameters for 2D Bioprinting of graded concentration of cells....11

Table S4. Flow rate parameters for 3D Bioprinting of graded concentration of cells....11

Figure S6. Physico-chemical analysis of the soft carboxylated agarose (CA)....12

Figure S7. Microscopic picture of LIVE/DEAD assay....13

Materials and Methods

Carboxylated agarose

Carboxylated agarose was synthesized as previously reported.[14] One gram of native agarose (NA) type 1 (GeneON, Germany) was transferred into a three-necked round bottom flask, equipped with a mechanical stirrer and pH meter. The reactor was heated to 90° to dissolve the agarose in water (500 mL) and then cooled to 0 °C under mechanically stirring to prevent the solution for gelling. The reactor was then charged with TEMPO (0.160 mmol, 20.6 mg), NaBr (0.9 mmol, 0.1 g), and NaOCl (2.5 mL, 15% vol/vol solution) under vigorous stirring. The pH of the solution was adjusted to pH 10.8 throughout the reaction, and the degree of carboxylation was controlled by the addition of predetermined volumes of NaOH solution (0.5 M). At the end of the reaction, $NaBH_4$ (0.1 g) was added, and the solution was acidified to pH 8 and stirred for 1 h. The CA was precipitated by sequential addition of NaCl (12 g) and ethanol (500 mL), and the solid was collected by vacuum filtration and extracted using ethanol. Residual ethanol was removed by extensive dialysis against water and the CA was obtained as a white solid upon freeze-drying overnight. In this study, CA of three different degrees of carboxylation were used to access hydrogels from 2 to 8 % w/v. Final optimized formulation of CA hydrogel were selected with 6% w/v with shear modulus at 1Hz of 1520 ± 106 Pa (soft), 2234 ± 149 Pa (medium) and 3745 ± 93 Pa (stiff). TEMPO, NaBr, NaOCl, NaCl and $NaBH_4$ were obtained from Sigma Aldrich (Germany) and used as received.

3D bioprinter

The bioprinter was costumed build and based on a Magician Robotic arm (DOBOT, China). A custom-built mixing head with two openings was machined from stainless steel (**SI-Figure 1**). Two openings (10-32 UNF) of 4.1 mm and 2 mm inner diameter channels at an angle of 135 ° with respect to one another converged into a 2.5 mm diameter channel of 7 mm length. In this channel a 6-elements static mixer with 15 mm length and 1.5 mm diameter static mixer was positioned (Misch- und Dosiertechnik GmbH, Germany) (**SI-Figure 5**). The mixing head was fitted with a barbed adapter on the feeding inputs and a Luer-lock at the output (Cole Parmer, USA). Two holes of 4 mm diameter each were drilled on the top of the head to accommodate a PT100 thermal sensor (Conrad, Germany) and a heating cartridge (Conrad, Germany). The heating system was controlled by a Thermo controller STC100. The tubes (4x2, polyurethane tubing, Cole Parmer, USA) carrying the bioink from the syringes to the mixing head were enfolded into a heating pad (New Era, USA). The temperature controller for the heating pads consists of a primary and secondary output. The primary exits are connected to cover the syringes, and the secondary outputs are connected to cover the pipes. The 10 mL syringes (Luer lock tip, Terumo, Japan) were kept at 40°C by two heating pads controlled by the New Era controller. The syringes were loaded with the printing media and positioned on the syringe pumps (Cole Parmer, USA). On the printing area below the arm of the robotic arm, a water-cooled stage was positioned as described earlier.[15] The custom-built stage was built of poly(carbonate) (PC) receptacle with two openings equipped with a tube connector to let the water flow through. On the PC bottom was glued an aluminum plate that was machined with threads to hold petri dish of different sizes. Cold water was circulated at 4°C through the printing stage to allow rapid gelation of the CA bioink.

Bioprinting design

The printed design was drawn on Inventor (Autodesk, 2018) and exported as an STL file. The files were then converted into G-code using Repetier Host (Hot-World GmbH) and Slic3R (Open Source). Printing parameters of the robot such as layer thickness and speed were controlled on Repetier Host. Two designs were created: (1) 2D serpentine line and (2) a 3D cylinder (**SI-Figure 2**).

3D bioprinting optimization

Various concentration of CA soft, medium, and stiff were prepared in phosphate-buffered saline solution (Gibco) ranging from 2% w/v to 8%w/v. Each formulation was printed, and the dimension of

the printed object was measured and compared with the dimension of the designed object. For the planar object, a picture of the object was taken with a reference dimension that allowed us to convert pixel into metric units. Using ImageJ (NIH, Bethesda, Maryland, USA) the diameter of the line was measured in pixel and then converted into a metric value. Each measurement is the average of nine printed samples and error bars represent the standard deviation. In case of the cylinders, the dimensions were measured with calipers. The outer diameter, inner diameter, and height of printed samples (n = 9) were measured, and the dimensions were compared with the input design to determine the deviation from the prescribed dimensions.

Maleimide functionalized agarose

Soft CA was dissolved in 0.1 M 2-(N-morpholino)ethanesulfonic acid buffer solution (pH 4) (Sigma Aldrich, Germany) in a round bottom flask by heating in a water bath at 90°C for 5 minutes under mechanical stirrer in a laminar flow. The maximum concentration of CA in the MES solution was 0.5 w/v% to avoid gel formation. Once the CA was dissolved the solution was cooled down to room temperature, and a 10-fold molar excess of EDC (Sigma Aldrich, Germany) dissolved in MES solution was added to the main solution to activate the carboxylic groups in the CA. After 45 minutes of activation, maleimide (1 mmol/g CA) was dissolved in MES solution and added to the main solution, and the solution could react for 24 hours at room temperature under mechanical stirring. The functionalized CA was purified by dialysis using a cellulose acetate membrane (3.5 kDa, MWCO, Spectra Pore, USA) against distilled water for three days. The dialysis waster was changed twice a day at 6-hour intervals. Finally, the sample was freeze-dried using liquid nitrogen in a round bottom flask and dried for 24 hours under vacuum (0.1 mbar).

^{1}H-NMR spectroscopy

^{1}H-NMR spectra were recorded at 25 °C, with water suppression on an Advance spectrometer at 300 MHz (Bruker, Germany). Samples' solutions (10 mg/ml) were prepared with deuterium oxide (D_2O) by heating in a water bath at 90 °C until a clear solution was obtained. All chemical shifts are reported in ppm relative to D_2O (δ = 4.8 ppm).

FTIR spectroscopy

Fourier transformed infrared (FTIR) spectrometer Vector 22 (Bruker, Germany) was used to record the IR spectra. Samples were mixed with potassium bromide (0.25 w/w%) and then pressed for 2 minutes at 10 ton into discs. All samples were measured using 30 scans with a resolution of 4 cm^{-1} in the 4000-600 cm^{-1} range.

CD spectroscopy

Circular dichroism (CD) spectra of functionalized carboxylated agarose (0.5 mg/ml) in a wavelength range of 180 to 300 nm were recorded on a J-810 CD spectrometer (Jasco, Japan). Measurements were carried out in a 1cm path length quartz cuvette at 25°C and the spectra is an average of four scans.

UV-Vis spectroscopy kinetic

UV-Vis measurements were collected using a Thermo Evolution 201 spectrometer (ThermoFischer, Germany). Quartz cuvettes were used for all samples, and the recorded absorbance was from 800-190 nm and the baseline was corrected at 750 nm. The kinetics of the thiol-ene reaction was performed with a 5-fold molar excess of CA-Maleimide (6 mg/ml). CA-Maleimide was dissolved in distilled water and the cysteine peptide (CRGDS, 593.61 g/mol, 98.82%, Proteogenix, France) was dissolved in PBS (0.2 mg/ml). Measurements were recorded at 37 °C with a magnetic stirrer for one hour.

Rheological measurements

Rheology experiments were performed with a Kinexus Pro rheometer (Malvern Panalytical, UK). Samples were dissolved in distilled water (2 w/v%) in a water bath at 90 °C for 5 minutes until a clear solution was obtained. All samples were stored at 4 °C for 24 h to allow gel formation before the measurements. A cone disc of 40 mm and 1° was used. The test was performed at 1 Hz shear frequency and room temperature. Each point is a mean of the measurement of three different experiments.

Bioprinting of stiffness gradient

The objects with graded mechanical properties were prepared by dissolving each of the soft, medium, and stiff CA in 10 ml syringes at 6% w/v. The syringes and tubes are wrapped in heating pads and the cold water is circulating through the cold stage. A static mixer (SI-Figure 5) was added in the mixing head before the extrusion nozzle. The static mixer was a polypropylene 6-elements Helix Mixer K-System 0001-9998-0208MKHX (Misch- und Dosiertechnik GmbH, Germany) with a length of 15 mm and diameter of 1.5 mm. The extrusion flow rate of each syringe is varied during the printing and set according to SI-Table 1 for the 2D printing and SI-Table 2 for the 3D printing. The mechanical properties of the printed object were determined using a Texture Analyzer TA-XT2i (Stable MicroSystems Ltd., Surrey, UK). The analyzer was equipped with a self-made cylinder probe (2 mm thickness, 3.14 mm^2 area). The measurement was started at a pre-force of 0.005 N and conducted at a speed of 0.5 mm/s till a maximum compressive strain of 20%. The indentation elastic modulus was calculated in the linear regime (ca. 2-6% strain). The 3D hydrogel samples were sliced parallel to z-axis (from bottom to top) and measurements were conducted in the z-axis direction, average values of hardness and modulus were calculated from three independent slices of a single hydrogel sample

Bioprinting of peptide gradient

The objects with graded mechanical properties were prepared by dissolving the soft CA in 10 ml syringes at 6% w/v and the CRGDS-FITC peptide at 1 mg/mL in PBS (Gibco, Germany). The syringes and tubes are wrapped in heating pads and the cold water is circulating through the cold stage. The extrusion flow rate of each syringe is varied during the printing and set according to SI-Table 3 for the 2D printing and SI-Table 4 for the 3D printing. A static mixer (SI-Figure 5) was added in the mixing head before the extrusion nozzle. The static mixer was a polypropylene 6-elements Helix Mixer K-System 0001-9998-0208MKHX (Misch- und Dosiertechnik GmbH, Germany) with a length of 15 mm and diameter of 1.5 mm. Peptide attachment was tested by printing with peptide at the same concentration a soft hydrogel and a soft hydrogel functionalized with maleimide. Each of the object (Soft CA and Soft CA-maleimide) printed with peptide was submerged in deionized water for 24h and the supernatant was collected at a different time interval and UV-vis spectrometry was performed to measure the amount of peptide diffused outside of the printed object. The gradient of peptide immobilized on the hydrogel was measured using ImageJ image analysis software. Photography of the sample was acquired, then converted in a black and white picture and the pixel intensity was calculated.

Cell concentration gradient bioprinting

As a proof of concept toward the fabrication of objects with cell gradients, HEK293 cells were printed. HEK293 were cultured in DMEM supplemented with 10% (vol/vol) FBS and 1% Penicillin-Streptomycin-Amphotericin B mixture in a humidified incubator at 5% CO_2 and 37°C. Cell viability was assessed by adding Hoechst 33342 (1µL/mL). Cells were trypsinized and loaded in 10 ml syringes at a concentration of 4 x 10^6 cells / mL. The cells solution was mixed in the printer with the CA soft formulation according to the flow rate sequences described in SI-Table 3 and SI-Table 4. A static mixer (SI-Figure 5) was added in the mixing head before the extrusion nozzle. The static mixer was a polypropylene 6-elements Helix Mixer K-System 0001-9998-0208MKHX (Misch- und Dosiertechnik GmbH, Germany) with a length of 15 mm and diameter of 1.5 mm. The incorporation of the cells in the 2D printed object was directly

assessed on a microscope Axio Vision (Zeiss, Germany) with a 10x objective. Three samples of the printed 3D objects were placed on a microscopy slide and pictures of the side of the cylinder were imaged and stitched together to form a complete image. The number of cells per field of view was counted using Image J. At each position, pictures of at different focal planes were acquired, the file was then turned into 8-bit grayscale, threshold and then the particles analyzer tool of ImageJ was used to count the number of cells.

Cell viability

HEK293 cells suspended at the concentration of 4 x 1 cells / mL in DMEM with 10% vol/vol FBS and 1% Penicillin-Streptomycin Amphotericin B mixture were mixed with the CA soft at different flow rate from 0.1 to 0.12 mL/min. In total 100 µL was extruded in a 96-wellplate (Nunc, Germany). The cell viability was then directly assay. We used a Live/Dead Assay kit (Life Technologies, Germany) immediately after printing. The staining solution was created by adding 0.2 µL calcein AM (Component A) and 1 µL ethidium homodimer-1 (Component B) into 1 mL DPBS. Each sample was stained with 100µL of the staining solution then incubated 30 minutes at 20–25°C. Optical microscopy images were obtained on a Zeiss Observer A1 (Carl Zeiss, Germany). The results were analyzed by Image J. The number of cells per field of view was calculated. The calculated percentage of living cells was normalized to a sample that was not bioprinted. Statistical analysis was done using Graphpad unpair t-test calculator (GraphPad Software, USA).

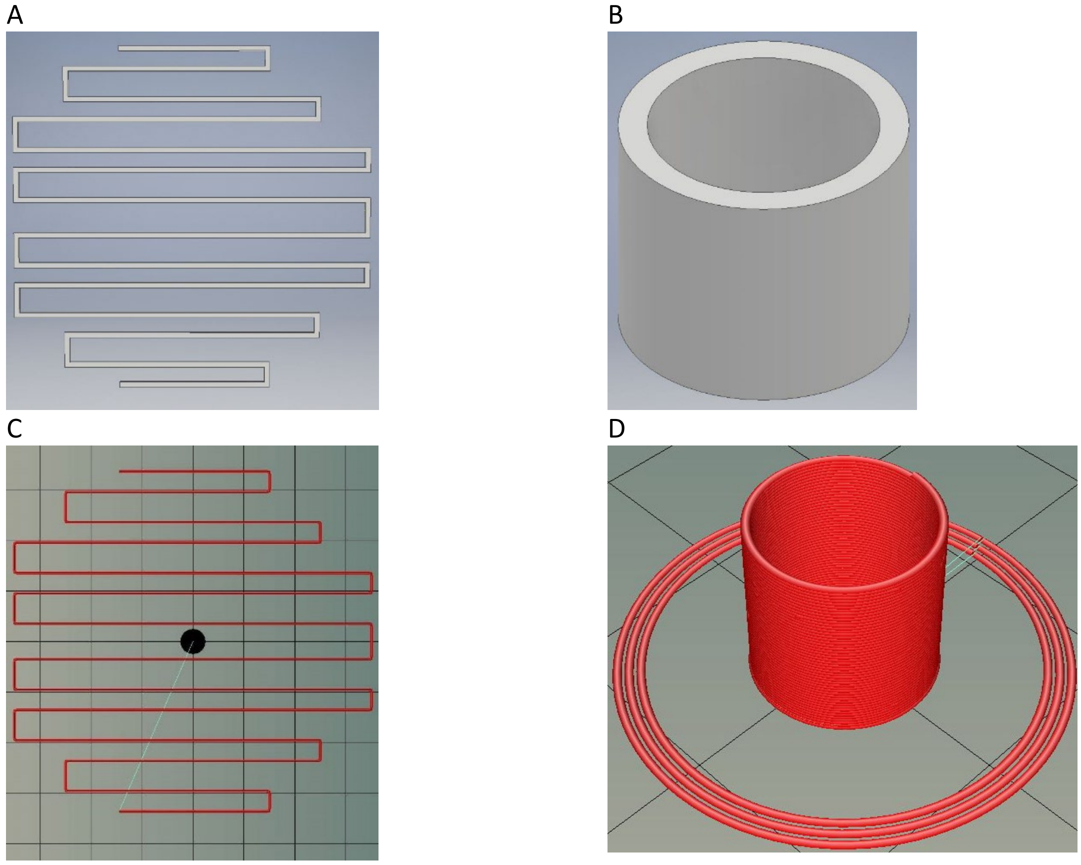

Figure S1. CAD of the printed objects. (**A**) Computer-aided-design of the printed 2D pattern. (**B**) Computer-aided-design of the printed 3D pattern. (**C**) Graphic representation of the GCode used to print the 2D pattern. (**D**) Graphic representation of the GCode used to print the 3D pattern

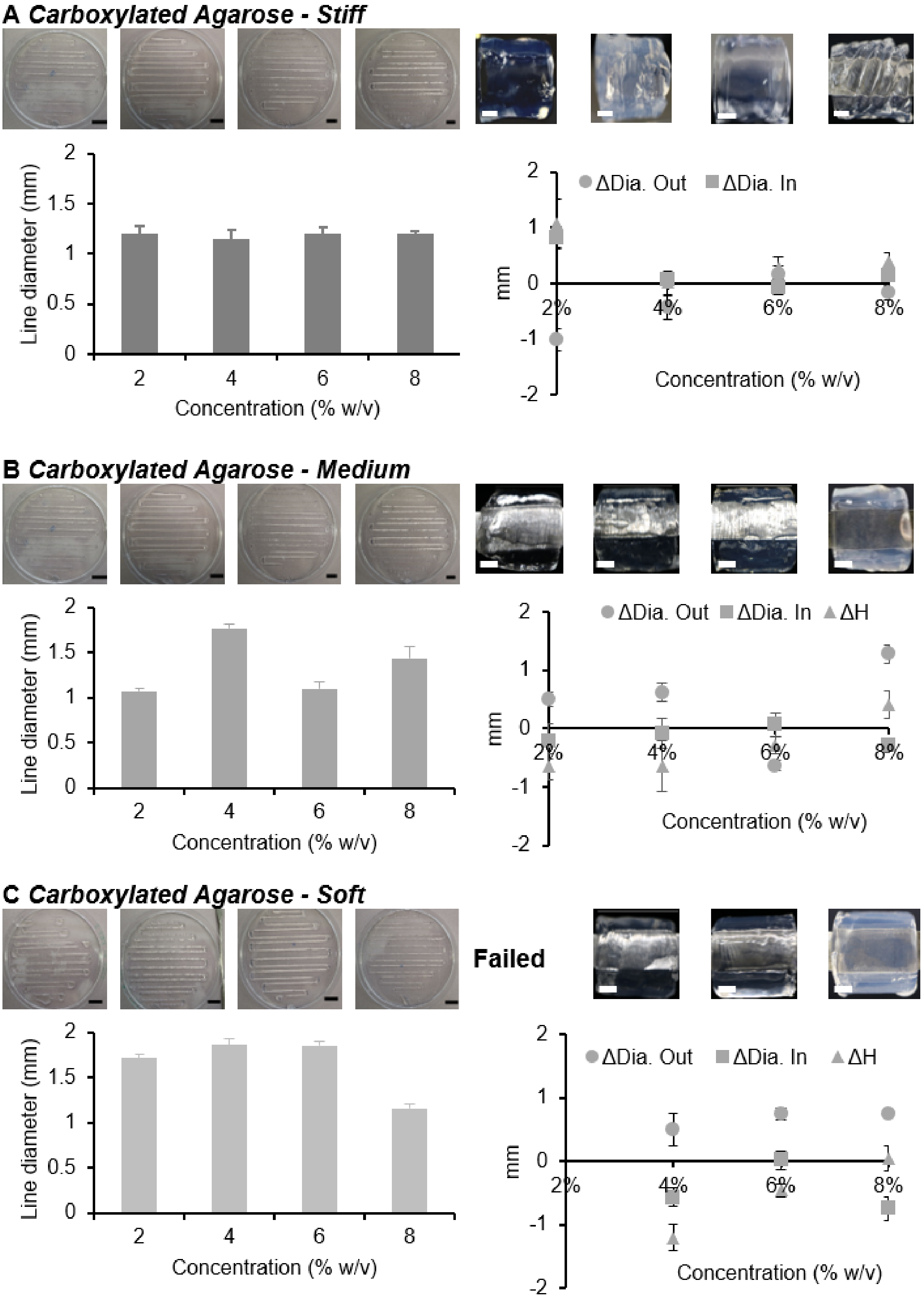

Figure S2. Optimization of printing parameter. The printing parameters were optimised for 2D serpentine lines (*left)* by measuring the line diameter, and for the 3D cylinders (*right)* by computing the difference between the dimension of the CAD file and printed object for the cylinders outer (*Δd(out)*) and inner (*Δd(in))* diameter and height (*Δh*), for different concentration of (**A**) stiff, (**B**) medium and (**C**) soft carboxylated agarose. Error bars represent standard deviation for n = 9. Scale bar: 10 mm (left panels) and 2 mm (right panels)

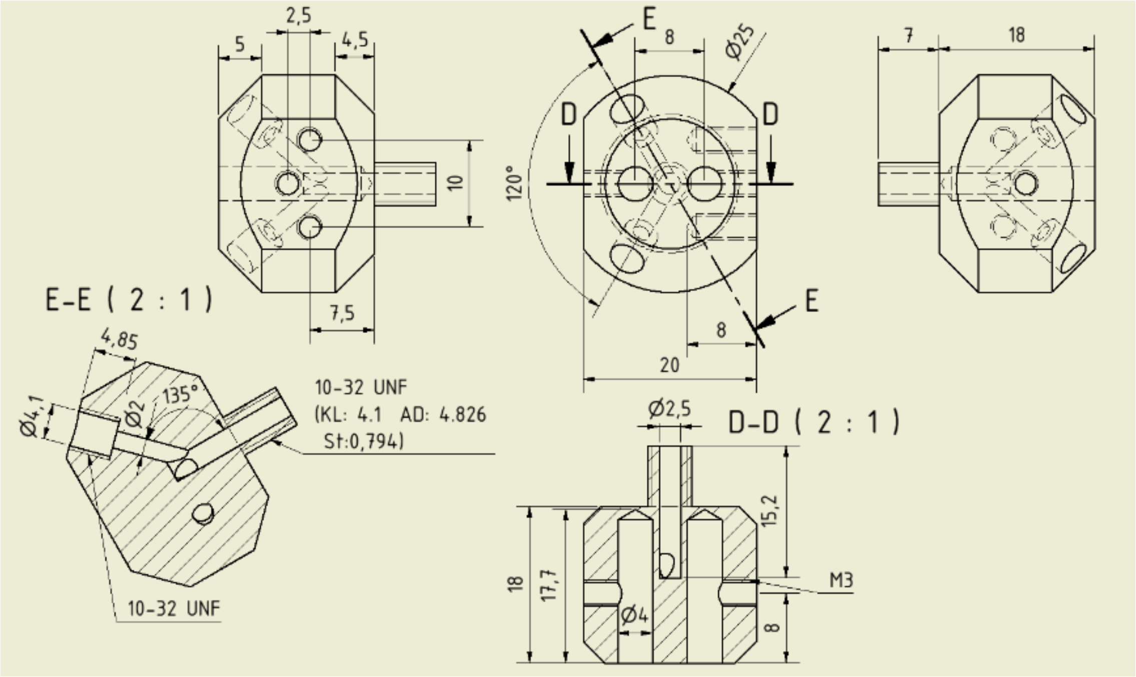

Figure S3. Technical drawing of the mixing head.

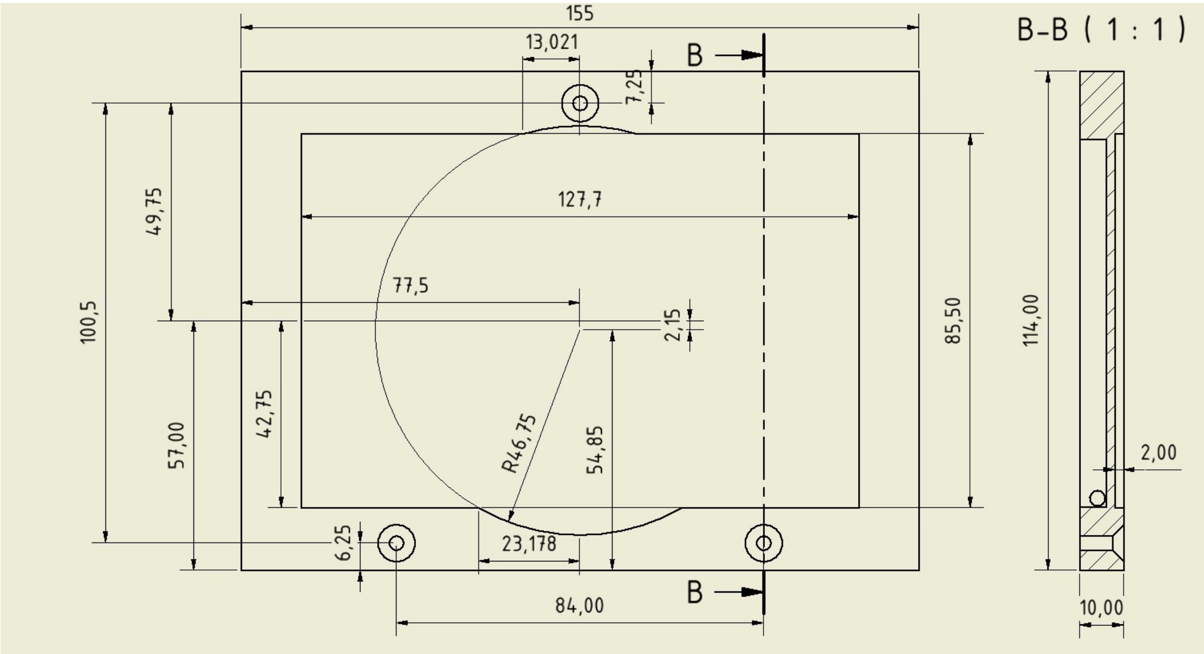

Figure S4. Technical drawing of the cooling stage.

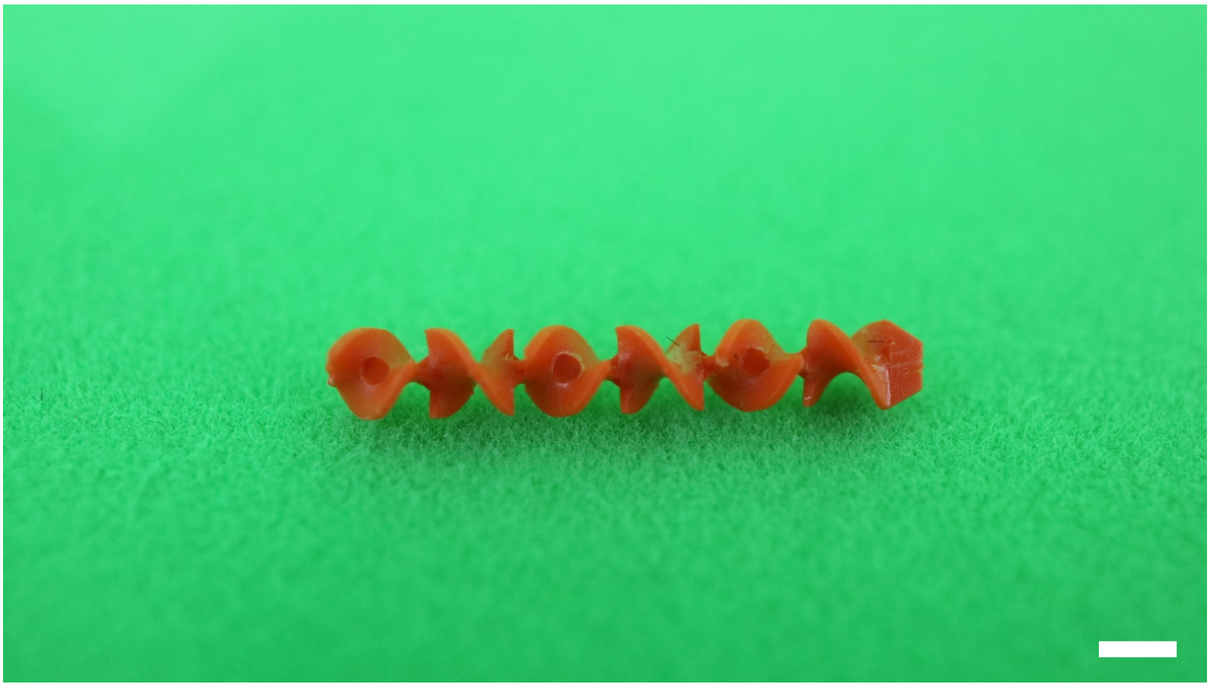

Figure S5. Static mixer. Picture of the mixing element used in this study. Scale bar 2 mm.

Table S1. Flow rate parameters for 2D Bioprinting of graded mechanical properties

Distance from the starting point (mm)	Stiff or Medium CA	Soft
	Flow Rate (ml/min)	
0	0.2	0
75	0.1	0.1
426	0	0.2

Table S2. Flow rate parameters for 3D Bioprinting of graded mechanical properties

	Stiff CA	Soft
Layer	Flow Rate (ml/min)	
0-16	0.2	0
17-32	0.1	0.1
33-50	0	0.1
	Medium CA	Soft
0-16	0.1	0
17-32	0.05	0.05
33-50	0	0.1

Table S3. Flow rate parameters for 2D Bioprinting of graded concentration of cells.

	Soft CA	HEK293 / Peptide
Distance from the starting point (mm)	Flow Rate (ml/min)	
0	0.2	0
75	0.15	0.05
279	0.1	0.1
503	0.05	0.15

Table S4. Flow rate parameters for 3D Bioprinting of graded concentration of cells.

	Soft CA	HEK293 / Peptide
Layer	Flow Rate (ml/min)	
0-10	0.1	0
11-20	0.09	0.01
21-30	0.08	0.02
31-40	0.07	0.03
41-50	0.06	0.04

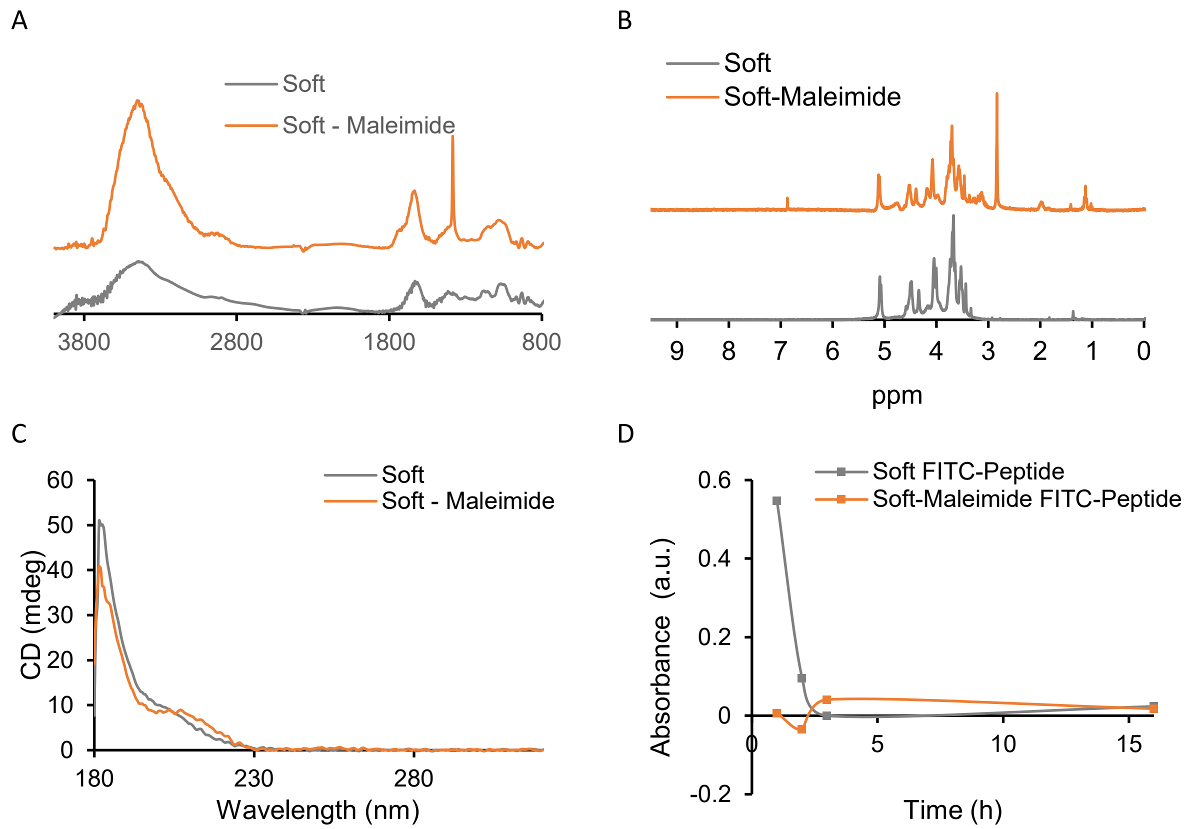

Figure S6. Physico-chemical analysis of the soft carboxylated agarose (CA). CA used in this study compared to the maleimide functionalized soft CA. (**A**) FTIR, (**B**) NMR, (**C**) Circular dichroism. (**D**) UV absorbance of water incubated with bioprinted object made of CA with FITC-peptide and maleimide functionalized CA with FITC-peptide for different incubation time.

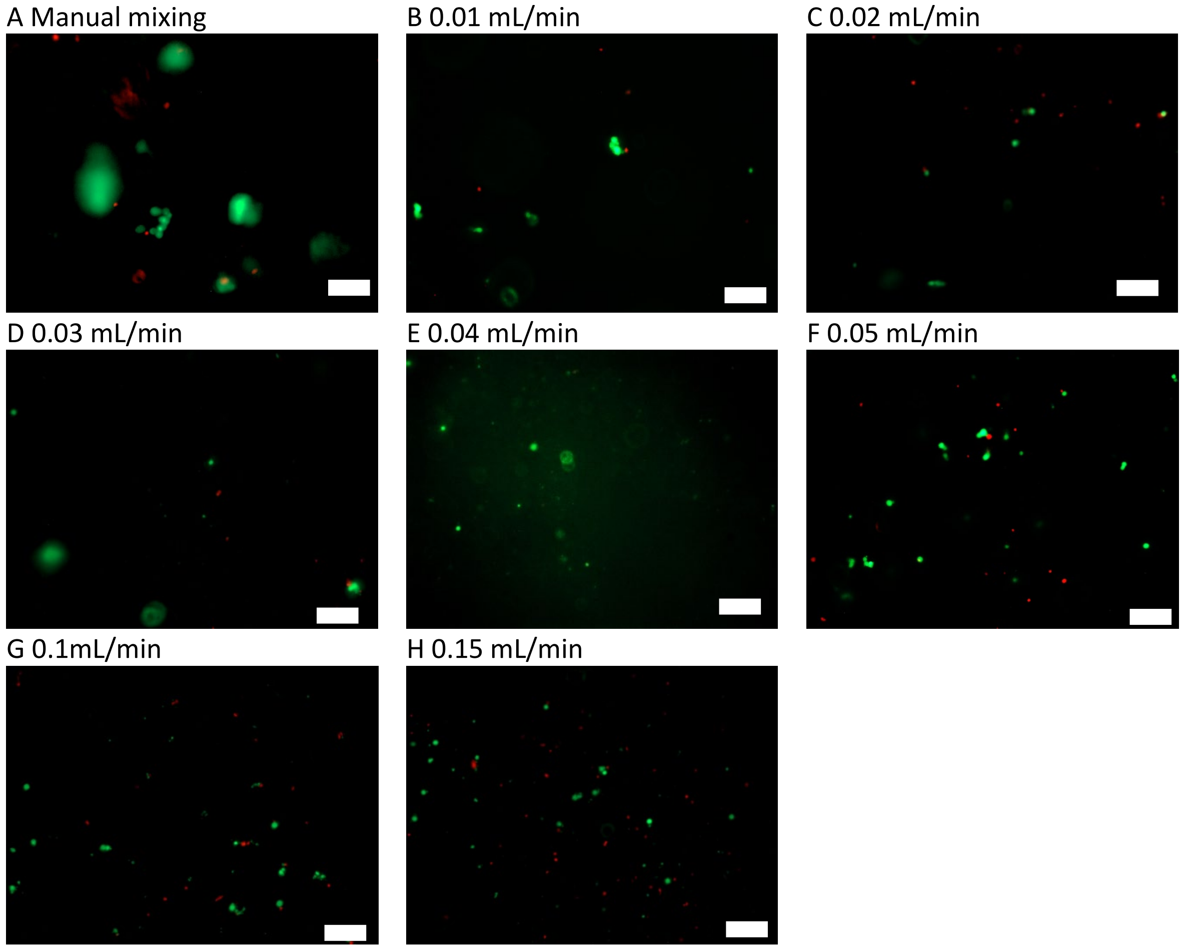

Figure S7. Microscopic picture of LIVE/DEAD assay. Representative images of the LIVE/DEAD assay performed on the CA hydrogel at different extrusion speed. An increase of cell death is observed with an increase of flow rate. While some dead cells are observed when the hydrogel are manually prepared as the CA lacks adhesion motifs. Scale bar 100 µm.
